# Maxillo-Facial Morphology in Beckwith-Wiedemann Syndrome: A Preliminary Study on (epi)Genotype-Phenotype Association in Caucasians

**DOI:** 10.3390/ijerph19042448

**Published:** 2022-02-20

**Authors:** Patrizia Defabianis, Alessandro Mussa, Rossella Ninivaggi, Diana Carli, Federica Romano

**Affiliations:** 1 Department of Surgical Sciences, C.I.R. Dental School, University of Turin, 10126 Turin, Italy; rossella.ninivaggi@unito.it (R.N.); federica.romano@unito.it (F.R.); 2 Department of Public Health and Pediatric Sciences, University of Turin, 10126 Turin, Italy; alessandro.mussa@unito.it (A.M.); diana.carli@unito.it (D.C.); 3 Pediatric Clinical Genetics, Regina Margherita Children Hospital, Città della Salute e della Scienza di Torino, 10126 Turin, Italy

**Keywords:** Beckwith-Wiedemann syndrome, Caucasian, ethnicity, imprinting disturbance, macroglossia, malocclusion, molecular testing, tongue reduction

## Abstract

Beckwith–Wiedemann syndrome (BWS) is a congenital overgrowth disorder caused by various (epi)genetic alterations affecting the expression of genes on chromosome 11p15. Cardinal features include abdominal wall defects, macroglossia, and cancer predisposition. Several (epi)genotype–phenotype associations were described so far, but specific studies on the evolution over time of maxillo-facial phenotype in the molecular subtypes still are scanty. The aim of this cross-sectional study was to associate maxillo-facial morphology and growth pattern with genoype in 25 Caucasian children with BWS and macroglossia. Twelve patients experienced a loss of metilation at imprinting center 2 (IC2-LoM), five had mosaic paternal uniparental isodisomy of chromosome 11 (UPD(11)pat), and eight were negative. A more marked tongue enlargement was detected in patients with IC2-LoM and negative genotype, while UPD(11)pat children showed mild macroglossia (*p* = 0.048). A cluster analysis did not demonstrate any specific relationship between (epi)genotype and maxillo-facial phenotype, but separated BWS patients based on their cephalometric characteristics. Children with IC2-LoM or negative genotype displayed hyperdivergence values > 30°, clockwise growth tendency, and skeletal class II into the same cluster. They had a negative prognostic score. These preliminary data suggest the need for developing individualized protocols for early monitoring of the craniofacial growth in such patients.

## 1. Introduction

Beckwith–Wiedemann Syndrome (BWS) (OMIM#130650) is the most common congenital overgrowth disorder in infancy, with an estimated incidence approximating 1 in 10,000 live births [1,2,3]. Macroglossia, somatic and lateralized overgrowth, hyperinsulinism, abdominal wall defects and cancer predisposition are its main features, to which other minor and less common features can be associated [4]. 

Macroglossia, found in 80–97% of the patients, is a phenotypically heterogeneous condition with different degrees of severity [5]. An enlargement of the tongue would seem to result from the hyperplasia of muscle fibers and is generally evident in all three dimensions [6]. As a consequence of its size, the tongue is interposed between the dental arches and the lips. This may lead to malocclusion, resulting in mandibular prognathism, anterior open bite, cross bites, and a wide dental arch. In addition, 48% of BWS patients suffer from sleep-disordered breathing that could negatively impact their skeletal growth pattern [7].

The molecular bases of BWS are complex. Epigenetic and genetic defects alter the expression of imprinted genes on chromosome 11p15.5, including insulin-like growth factor 2 (*IGF2*), the long noncoding RNA *H19*, and cyclin-dependent kinase inhibitor 1C (*CDKN1C*), which control fetal and postnatal growth and cell proliferation [8,9]. Five molecular alterations have been commonly related to BWS, including loss of methylation at imprinting center 2 (IC2-LoM), occurring in 50% of cases; mosaic paternal uniparental isodisomy for part/all of chromosome 11 (UPD(11)pat) in 20–25%; gain of methylation at imprinting center 1 (IC1-GoM) in 5–10%; maternally inherited inactivating mutations of *CDKN1C* in 5–10%; and chromosomal rearrangements in <1% [4,8,9,10]. Approximately 20% of clinically diagnosed BWS patients lack a definite (epi)genotype. In 2018, the International BWS Consensus group established recommendations for its diagnosis and management and introduced the definition of Beckwith–Wiedemann spectrum (BWSp), including patients with typical features and epigenetic anomalies, few features and epigenetic anomalies (“atypical BWS”) and typical features but negative molecular tests [11]. Within the broad spectrum of clinical features, it has become increasingly evident that there is a strict relationship between (epi)genotype and phenotype [8,12,13,14,15]. Some of these associations have also been evidenced in the prevalence and severity of macroglossia and BWS-related facial features [11].

Indications for tongue reduction surgery (TRS) have not been clearly defined. While some studies recommend early surgical treatment of macroglossia to prevent mandibular prognathism and open bite, others demonstrated that TRS does not control the pattern of mandibular growth and the development of class III skeletal malocclusion [16,17,18,19]. Indeed, the enlarged mandibular body observed in BWS might be caused by mandibular cartilaginous growth activated by *IGF-2* expression, rather than being secondary to tongue overgrowth [20]. Evolution over time of mandibular growth and dental issue is of concern and represents a major health problem of patients with BWS in adulthood too [21]. Consistent with recommendations and guidelines for clinical management and tumor surveillance strategies, it would be useful to develop protocols for monitoring the craniofacial growth in these patients according to race/ethnicity. While a racial/ethnic predisposition for BWS has not been reported as yet [4], a role for race/ethnicity in molecular subtypes distribution and clinical features expression was suggested in small cohorts of patients from Japan [22] and China [23].

The aim of this cross-sectional study was to associate maxillo-facial morphology with the underlying molecular defects in Caucasians. Such relationship would allow for a more accurate prediction of growth patterns and long-term prognosis of the orthodontic-orthopedic treatment according to (epi)genotype, as well as for an individualized treatment strategy. Moreover, the real need for invasive TRS could be assessed based on the different biological background of the different epigenotypes.

## 2. Materials and Methods

### 2.1. Study Design

All patients recruited for the study were followed by the same pediatric geneticist at the Regina Margherita Hospital and were consecutively referred to Section of Pediatric Dentistry, C.I.R. Dental School, University of Turin (Italy) between September 2019 and October 2021.

All patients were diagnosed with BWSp according to the 2018 criteria [11] and underwent Methylation-Specific Multiplex Ligation-dependent Probe Amplification (MS-MLPA, © SALSA MLPA Probemix, MRC Holland, the Netherlands) for 11p15 abnormalities on DNA extracted from peripheral blood (*n* = 25) and skin biopsy of the affected overgrown region (*n* = 2) [24] UPD(11)pat was confirmed using an SNP array or microsatellite segregation analysis [25] and patients who tested negative in these analyses were also submitted to *CDKN1c* sequencing [26] and Chromosomal Microarray Analysis (CMA), according to the current protocols [11].

Exclusion criteria included history of previous TRS and/or previous orthodontic treatment, since they could affect the growth trends. In addition, patients younger than 4 years and those not cooperating in the execution of the radiographic documentation were also excluded. Ethical approval to participate was granted by the Institutional Ethics Committee (1103-2019, Città della Salute e della Scienza di Torino) and written informed consent was obtained from parents or legal guardians. The investigation was performed according to the ethical principles of the Helsinki Declaration.

### 2.2. Data Collection

A single specialist in pediatric dentistry performed the clinical examinations. He recorded Angle’s dental class and the presence of any carious lesion using the decayed, missing and filled teeth index (dmft in primary and DMFT in permanent dentition). All patients were asked about phonation difficulties and speech therapy. Cast models, intraoral and facial photographs were taken to analyze oral and facial appearance.

Orthopantomography of the dental arches and lateral cephalometric radiograph of the skull were performed for all patients to assess the presence of dental agenesis and anomalies and to evaluate the skeletal malocclusion and growth tendency.

Traditional cephalometric landmarks (Table 1) were used according to the architectural and structural analysis proposed by Björk-Jarabak [27,28]. In a dark room, a single experienced clinician, blinded to the (epi)genotype, manually performed each cephalometric analysis by tracing the craniofacial landmarks and linear parameters with transparent 0.003” matte-acetate paper and a graphite pencil (point 0.3). Measurement were obtained with the aid of a millimeter ruler and a 360° protractor, and angular assessments were approximated to 0.5° and linear measurements to 0.5 mm. In order to verify the accuracy of landmarks, a second investigator checked the data. The same clinician repeated each measurement twice, with a 4-week interval in between, to minimize errors. 

The following parameters were recorded: (1) intermaxillary divergence (SpP^GoGn) considering hypodivergence (<15°), normal divergence (20° ± 5°) or hyperdivergence (>25°), (2) skeletal class I (ANB < 0°), class II (ANB between 0° to 4°) and class III (ANB > 4°) jaw relationship; (3) vertical growth pattern based on the ratio between the posterior and the anterior facial height (<62% for clockwise growth with ante-rotation of the mandible; 62–65% in the case of straight-down growth; and >65% for counter clockwise growth with post-rotation of the mandible); (4) upper and lower lip profile.

Children were also assigned a positive/negative/neutral prognostic score based on the cranial growth pattern in relation to the intermaxillary divergence. In case of hyperdivergence, they were assigned a negative score if they had a counter clockwise mandibular rotation, a positive score for the clockwise rotation and finally, a neutral score for the straight-down growth tendency. In case of normovergence, the combination with counter clockwise, clockwise or straight-down growth tendency was classified as neutral, negative or positive, respectively.

The cephalometric parameters and the molecular subgroup of each patient were anonymized, included in a single database and subjected to statistical analysis.

### 2.3. Data Analysis

Data were analysed using statistical software (SPSS, version 25, IBM Corp., Armonk, NY, USA). Qualitative data were presented as absolute and relative frequencies and quantitative data as mean and standard deviation (S.D.) or median and interquartile range (IQR), as appropriate. The Shapiro–Wilk test and Q-Q normality plots were applied to verify the normal distribution of quantitative variables.

Fisher’s exact test was used to evaluate any potential association between categorical variables and the ANOVA (for normally distributed variables) or the Kruskal–Wallis test (for non-normally distributed variables) was used to assess differences of quantitative variables among (epi)genotypes. A multivariate exploratory data analysis was performed using hierarchical cluster analysis to assess whether it is possible to discriminate BWS (epi)genotypes based on the similarity of skeletal divergence, cranio-facial growth pattern, skeletal class and facial profile. Clustering was based on the squared Euclidean distance and clusters were merged based on the Ward’s hierarchical method. Results were reported in the form of a dendrogram. Statistical significance was set at 5% for all analyses.

## 3. Results

### 3.1. Study Participants and Intraoral Characteristics

A total of 46 Caucasian children were considered for enrollment, 14 were excluded because they did not meet the inclusion criteria, and seven refused to participate. Finally, 25 children (10 boys, 15 girls) between 4 and 11 years of age (mean 6.7 ± 2.1 years) were included in this cross-sectional study. Twelve patients (48.0%) had IC2-LoM and five patients (20.0%) had UPD(11)pat. The methylation level of the chromosome 11p15.5 region, *CDKN1C* gene sequencing and SNP-array analysis were normal in the remaining 8 (32.0%) patients, who received a clinical diagnosis of BWSp (2018 diagnostic score > 4). None presented methylation gain in IC1 1 (IC1-GoM).

The demographic and clinical findings of the patients according to the molecular subtype are summarized in Table 2. The three groups were balanced for age and gender. None showed tooth agenesis, and there was no significant change in caries experience and oral hygiene habits among them. No children had cleft palate, and only one, belonging to the UPD(11)pat subtype, was diagnosed with Wilms tumor.

Macroglossia was observed in all of the enrolled children, with moderate expression in both IC2-LoM and genotype negative groups, while the majority of UPD(11)pat children showed mild expression (*p* = 0.048). Consistently, all of the children followed by a speech therapist for phonation difficulties belonged to the IC2-LoM group.

### 3.2. Maxillo-Facial Morphology and Growth Pattern

As reported in Table 3, most of the children (76.0%) had a hyperdivergent phenotype and dental Class I relationship. About 40% had an anterior open bite and 16% showed also unilateral posterior cross-bite. Meanwhile, no statistically significant differences were observed among the different (epi)genotypes. However, when considering the skeletal class jaw relationships, all the children with UPD(11)pat displayed class I, and almost all children who were negative in genetic testing had a class II relationship (*p* = 0.007). 

Overall, the growth tendency was distributed as follows: clockwise (44.0%), straight-down (44.0%) and counter clockwise (12.0%) pattern. Clockwise growth pattern was observed only in IC2-LoM and genetic test negative groups, while UPD(11)pat was associated with straight-down growth tendency. However, this trend did not reach statistical significance. 

Comparisons of the mean values of the cephalometric parameters among the three groups are given in Table 4. No statistically significant difference was detected in any measurement. 

As summarized in Table 5, all children with UPD(11)pat had a neutral prognostic score, while unfavorable scores were observed only in children with IC2-LoM or negative tests groups.

**Figure 1:**
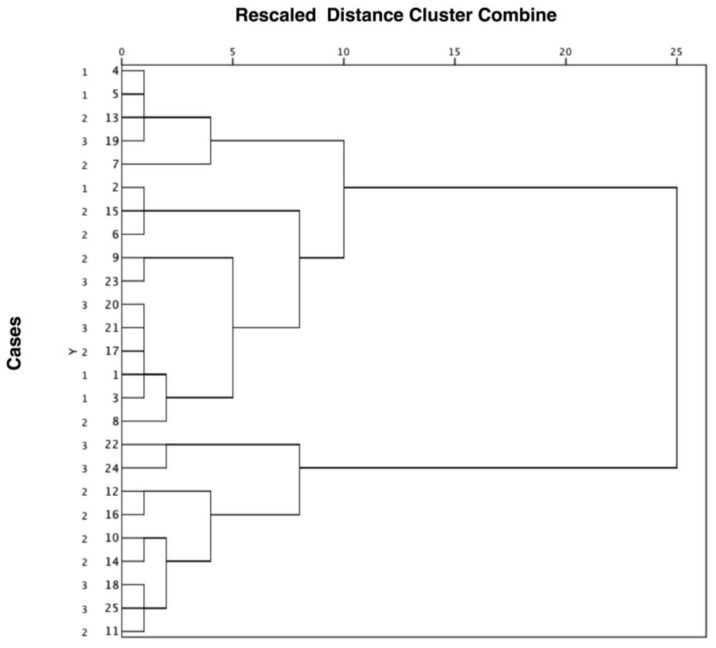
Clustering diagram showing that cases with Beckwith-Wiedemann syndrome were not separated on the basis of the molecular subtype (1 = UPD(11)pat; 2 = IC2-LoM; 3 = negative genetic tests) but on the severity of the cephalometric measurements. The distance level between cases or groups is measured along the horizontal axis, and the different cases with the corresponding molecular subtype are listed along the vertical axis.

The results of the hierarchical cluster analysis are presented in the form of a dendogram in Figure 1. It can be seen that the molecular subtypes clustered together, indicating that there is no association between maxillo-facial phenotypic features and BWS (epi)genotype. Interestingly, children with BWS were divided into two groups based on cephalometric measurements. All children with IC2-LoM or who were negative to genetic tests and displayed more severe hyperdivergence (values > 30°), clockwise growth tendency and skeletal class II were grouped together in the lower cluster. They also had a negative prognostic score (Appendix A).

## 4. Discussion

BWS is the most common pre and postnatal overgrowth disorder in infancy [1,2,3]. The phenotypic spectrum is highly variable, paralleling its (epi)genetic heterogeneity. Currently, (epi)genotypes can be grouped into five subtypes, besides the clinically diagnosed individuals who do not have a detectable molecular abnormality [9]. 

Recent studies evidenced relevant (epi)genotype–phenotype association [2,8,29,30] and clinical and epigenetic differences based on racial/ethnic background [22,23]. Caucasian patients are more likely to present with classic traits of BWS (macroglossia, omphalocele) along with IC2-LoM, while non-Caucasian and Asian patients appear to be more prone to show less visually apparent features (nephromegaly, hyperinsulinism) reflecting UPD(11)pat and IC1-GoM subtypes [31]. These findings may be reflective of specific differences or modifiers within different racial or ethnic groups that predispose persons to distinct epigenetic/genetic alterations.

Consistent with the findings in Western populations, nearly 50% of our cohort of Caucasian children had IC2-LoM, 20% had UPD(11)pat, and 30% were negative to genetic tests. All the enrolled children had untreated macroglossia of a variable degree. In agreement with the literature data, a more severe tongue enlargement was associated with IC2-LoM and showed no gender difference [2,8,31]. 

To the best of our knowledge, no previous study explored the association between (epi)genetic defect and maxillo-facial morphology/skeletal growth pattern in children with BWS-related macroglossia in depth. Tongue enlargement is the most common phenotypic trait in BWS [4,5]. Past studies reported that the increased pressure of the tongue results in anterior open bite and a prognathic mandibular appearance secondary to an abnormally obtuse gonial angle and increased mandibular length, which can lead to negative consequences in social acceptance, projection of self-image, and psychological well-being [20,32]. Indeed, TRS represents the second most common surgical intervention after repair of omphalocele in patients with BWS, and it is currently recommended to correct the obstruction of the upper airways, sleep apnea, feeding difficulties or language delay, and to prevent musculoskeletal and dentoalveolar malformations [33,34]. Taking into account the invasiveness of TRS, its impact on breathing, language and quality of life, it is paramount to define whether BWS individuals really benefit from this procedure to prevent dentofacial deformities [18,35,36].

Interestingly, our cohort showed no skeletal class III malocclusion. This leads us to hypothesize that the incidence of skeletal class III in patients with BWS is substantially lower than hitherto reported. Most of the data provided in the literature rely on single case reports or small case series involving a limited number of children [16,17,32,37]. Although macroglossia might stimulate mandibular growth, it is difficult to determine whether the prognathism of the mandibular basis is a result of macroglossia and dentoalveolar changes or if it is genetically driven [38]. In the present study, about 50% of the children had skeletal class II relationship and 76% of them had intermaxillary hyperdivergence. This is consistent with the finding of anterior open bites, which in 16% of the children occurred along with unilateral posterior cross bites. Skeletal class jaw relationships were significantly correlated with the molecular subtypes, with class I more common in patients with UPD(11)pat and class II more common in those with negative genetic tests.

With regard to the growth tendency, the clockwise and straight-down patterns were the most common, and were both detected in 44.0% of the BWS cases, while the counter clockwise tendency was observed only in a minority of cases (12.0%). It is worth noting that clockwise growth pattern was detected only in patients with IC2-LoM or negative genetic tests, while straight-down growth tendency mostly occurred in those with UPD(11)pat, although this trend did not reach statistical significance, likely due to the small sample size. 

Concerning the prognosis of functional orthodontic treatment, we found that 56% of children with BWS had a positive prognosis for open bite reduction with orthodontic treatment. This finding is relevant as it questions the need of TRS for orthodontic reasons. Consistently, other authors expressed concerns regarding the real benefit of early surgical treatment in preventing mandibular prognathism and open bite [19,38]. Naujokat et al. reported a similar occurrence of dentoalveolar and musculoskeletal malformations in BWS, irrespective of TRS. Malpositioned teeth and anterior open bites were observed in 62% and 58% of surgically treated children, respectively, versus 80% and 70% of those not treated by TRS [38]. Meazzini et al. did not find any positive influence of early TRS on mandibular growth pattern [19]. It should also be considered that the size of macroglossia decreases as the child grows up [39].

We also applied a cluster analysis to explore whether skeletal divergence, cranio-facial growth pattern, skeletal class and facial profile might differentiate between the diverse molecular subtypes of BWS. While bivariate (epi)genotype-phenotype association has been reported in the literature [2,8,22,23,29,30,31], this is the first study to use multivariate statistical methods to classify patients according to dentoskeletal pattern. Interestingly, children were not separated based on this composite outcome. However, all IC2-LoM or genetic test negative children displaying more severe hyperdivergence (values > 30°), clockwise growth tendency and skeletal class II were grouped together. They also had a negative prognosis with a tendency for the malocclusion to worsen over time. For this reason, these patients should be early screened to early detect odonthostomatologic issues to minimize complications and provide individualized treatment.

The present study has some limitations. First of all, the size of the sample population might have prevented some differences from reaching statistical significance. However, the rarity of the disease in the general population should be considered [1,2]. Taking into account that the (epi)genotype and phenotype may be different based on racial/ethnic factors, we enrolled only Caucasian children. However, this limits the external generalizability of the present findings to other populations [31].

Furthermore, due to the lack of a scoring system to objectively measure the severity of macroglossia, we used subjective criteria [17], introducing a potential source of bias. Some clinical and cephalometric features were proposed for the diagnosis of macroglossia [40], but they could be a direct consequence of tongue enlargement or may not be directly related to it. Finally, our population was mostly made up of children with IC2-LoM or UPD(11)pat and thus we cannot rule out that clinically and statistically significant association between dentoskeletal and (epi)genotipic patterns may be detected in the other molecular subtypes [4].

## 5. Conclusions

This is the first study correlating the maxillo-facial characteristics and growth patterns with molecular subtypes in Caucasian children. 

Because of the close relationship to the oral function and maxillo-facial morphology, BWS patients with macroglossia should enter a program of periodic dental and orthodontic visits early, with strict monitoring. The IC2-LoM subtype seems to be related to a worse skeletal growth tendency with a high risk for progressive deterioration of the malocclusion. This is aimed at facilitating the establishment of appropriate and individualized orthognathic–orthodontic and speech-treatment protocols, with a view of improving their quality of life. Given the rarity of BWS, multicenter studies with larger cohorts and including the most rare BWS (epi)genotypes are needed to confirm these findings. Further analysis with larger datasets is also needed to determine how race/ethnicity plays a role in the presentation of clinical features and the molecular diagnosis of patients affected by BWS. 

## Figures and Tables

**Table 1:** Angular and linear cephalometric parameters and their definitions.

Parameters	Description
SpP^GoGn (°)	Angle drawn by a line connecting Go and Gn to SpP plane
Ant-HT NMe (mm)	Anterior facial height
Post-HT SGo (mm)	Posterior facial height
Ls-E line (mm) (PogC-En)	Horizontal distance from E-line (line connecting tip of nose and soft tissue chin) to labrale superius
Li-E line (mm)	Horizontal distance from E-line to labrale inferius
SNA (°)	Angle between the SN plane and NA line, sagittal position of subspinale relative to cranial
SNB (°)	Angle between the SN plane and NB line, sagittal position of supramentale relative to cranial base
ANB (°)	Difference between SNA and SNB angles

**Table 2:** Demographic and oral characteristics of patients with Beckwith-Wiedemann syndrome (BWS) divided by (epi)genotype.

	Group				
Variables	UPD(11)pat (*n* = 5)	IC2-LoM (*n* = 12)	Genetic Test Negative (*n* = 8)	Total (*n* = 25)	*p*-Value
Age (years), mean ± S.D.	7.2 ± 2.3	5.8 ± 1.3	7.7 ± 2.7	6.7 ± 2.1	0.139
Sex, female/male	2/3	8/4	5/3	15/10	0.758
Agenesis, *n* (%)	0 (0.0)	0 (0.0)	0 (0.0)	0 (0.0)	-
Interdental spaces, n (%)	4 (21.1)	9 (47.4)	6 (31.6)	19 (76.0)	1.000
Macroglossia, *n* (%)					0.048
Mild	4 (44.4)	4 (44.4)	1 (11.1)	9 (36.0)	
Moderate	1 (6.3)	8 (50.0)	7 (43.8)	16 (64.0)	
dmft, mean ± S.D.	1.0 ± 1.4	2.7 ± 2.6	2.9 ± 2.7	2.4 ± 2.5	0.380
DMFT, median (IQR)	0.0 (1.50)	0.0 (1.50)	0.0 (2.75)	0.0 (1.0)	0.979
Professional oral hygiene frequency, *n* (%)					0.179
At least once/year	1 (6.7)	8 (53.3)	6 (40.0)	15 (60.0)	
Occasionally	4 (40.0)	4 (40.0)	2 (20.0)	10 (40.0)	
Phonation difficulties, *n* (%)	0 (0.0)	5 (100.0)	0 (0.0)	5 (25.0)	0.053
Atypical deglutition, *n* (%)	0 (0.0)	5 (55.6)	4 (44.4)	9 (36.0)	0.198

dmft, decayed missing filled primary teeth index; DMFT, decayed missing filled permanent teeth index; IC2-LoM, loss of methylation at imprinting center 2; UPD(11)pat, mosaic paternal uniparental isodisomy of chromosome 11; IQR, interquartile range; S.D., standard deviation.

**Table 3:** Maxillo-Facial Morphology and Growth Pattern According to the Beckwith-Wiedemann Syndrome (BWS) (epi)Genotype.

	Group				
Variables, *n* (%)	UPD(11)pat (*n* = 5)	IC2-LoM (*n* = 12)	Genetic Test Negative (*n* = 8)	Total (*n* = 25)	*p*-Value
Angle Class					0.328
I	5 (26.3)	9 (47.4)	5 (26.3)	19 (76.0)	
II	0 (0.0)	3 (50.0)	3 (50.0)	6 (24.0)	
III	0 (0.0)	0 (0.0)	0 (0.0)	0 (0.0)	
Skeletal Class					0.007
I	5 (41.7)	6 (50.0)	1 (8.3)	12 (48.0)	
II	0 (0.0)	6 (46.2)	7 (53.8)	13 (52.0)	
III	0 (0.0)	0 (0.0)	0 (0.0)	0 (0.0)	
Divergence					0.826
Normovergence	1 (16.7)	4 (66.7)	1 (16.7)	6 (24.0)	
Hyperdivergence	4 (21.1)	8 (42.1)	7 (36.8)	19 (76.0)	
Hypodivergence	0 (0.0)	0 (0.0)	0 (0.0)	0 (0.0)	
Growth pattern					0.136
Clockwise	0 (0.0)	6 (54.5)	5 (45.5)	11 (44.0)	
Straight-down	4 (36.4)	4 (36.4)	3 (27.3)	11 (44.0)	
Counter clockwise	1 (20.0)	2 (80.0)	0 (0.0)	3 (12.0)	

IC2-LoM, loss of methylation at imprinting center 2; UPD(11)pat, mosaic paternal uniparental isodisomy of chromosome 11.

**Table 4:** Comparison of cephalometric measurements according to the Beckwith-Wiedemann syndrome (BWS) (epi)genotype.

	Group			
Variables	UPD(11)pat (*n* = 5)	IC2-LoM (*n* = 12)	Genetic Test Negative (*n* = 8)	*p*-Value
SNA(°), mean ± S.D.	81.4 ± 1.9	81.7 ± 3.3	82.4 ± 3.4	0.844
SNB (°), mean ± S.D.	78.1 ± 1.4	76.1 ± 3.2	75.6 ± 2.2	0.234
ANB (°), mean ± S.D.	3.3 ± 1.2	5.7 ± 4.2	6.8 ± 3.9	0.179
SpPGoGn (°), median (IQR)	27.8 (4.2)	28.5 (7.1)	28.8 (11.6)	0.719
Post-HT (mm), mean ± S.D.	63.2 ± 6.6	66.2 ± 5.7	67.7 ± 5.9	0.432
Ant-HT (mm), median (IQR)	100.0 (18.9)	107.5 (10.2)	108.1 (15.0)	0.092
Ls-E line (mm), median (IQR)	−1.0 (2.5)	1.4 (4.0)	1.0 (1.7)	0.433
Li-E line (mm), mean ± S.D.)	0.6 ± 3.1	2.1 ± 3.6	1.3 ± 2.3	0.656

IC2-LoM, loss of methylation at imprinting center 2; UPD(11)pat, mosaic paternal uniparental isodisomy of chromosome 11; IQR, interquartile range; S.D., standard deviation.

**Table 5:** Prognostic score in children with Beckwith-Wiedemann syndrome (BWS) according to the (epi)genotype.

	Group				
Variable, *n* (%)	UPD(11)pat (*n* = 5)	IC2-LoM (*n* = 12)	Genetic Test Negative (*n* = 8)	Total (*n* = 25)	*p*-Value
					0.078
Positive	0 (0.0)	2 (100)	0 (0.0)	2 (8.0)	
Negative	0 (0.0)	6 (54.5)	5 (45.5)	11 (44.0)	
Neutral	5 (41.7)	4 (33.3)	3 (25.0)	12 (48.0)	

IC2-LoM, loss of methylation at imprinting center 2; UPD(11)pat, mosaic paternal uniparental isodisomy of chromosome 11.

## Data Availability

The data underlying this article cannot be shared publicly due to the vulnerable study population. The data will be shared on reasonable request to the corresponding author.

## Appendix A

We performed a multivariate exploratory data analysis to assess whether it was possible to discriminate Beckwith-Wiedemann syndrome (BWS) (epi)genotypes on the basis of the individual skeletal and growth pattern profile. We employed the hierachical cluster analysis (or hierarchical clustering) that is an approach of grouping together cases based on the similarity of response to several quantitative variables. We considered as grouping variables the intermaxillary divergence (SpP^GoGn), the skeletal class (ANB°), the vertical growth pattern based on the ratio between the posterior and the anterior facial height and the upper and lower lip profile (Ls-E line and Li-E line). Data were standardized before proceeding with the analysis. We used an agglomerative method. Clustering was based on the squared Euclidean distance (the geometric distance between two observations) and clusters were combined using the Ward’s method of linkage. This algorithm joins cases into clusters if there inclusion produces the least increase in the error.

Results of the cluster analysis were summarized graphically into the dendogram. This diagram displayed the groups that are formed by clustering BWS cases at each step and their distance level. The distance level is measured along the horizontal axis and the different BWS cases are listed along the vertical axis.

We interpreted the groups by analyzing their content. The different BWS (epi)genotypes were not grouped into different clusters suggesting that there was no association between the molecular subtype and the skeletal and growth pattern profile. Looking at the characteristics of BWS children, it appeared that they were clustered based on their cephalometric measurements. The final grouping was obtained by cutting the diagram at a distance level of approximately 10. The first cluster was composed of most the observations and the second cluster of 9 observations. BWS cases with IC2-LoM or negative to genetic tests who displayed more severe hyperdivergence (values > 30°), clockwise growth tendency and skeletal class II were grouped together in the lower cluster.
